# A High-Precision Time-Frequency Entropy Based on Synchrosqueezing Generalized S-Transform Applied in Reservoir Detection

**DOI:** 10.3390/e20060428

**Published:** 2018-06-03

**Authors:** Hui Chen, Yuanchun Chen, Shaotong Sun, Ying Hu, Jun Feng

**Affiliations:** 1 Geomathematics Key Laboratory of Sichuan Province, Chengdu University of Technology, Chengdu 610059, China; 2 State Key Laboratory of Oil and Gas Reservoir Geology and Exploitation, Chengdu University of Technology, Chengdu 610059, China; 3 ConocoPhillips School of Geology and Geophysics, University of Oklahoma, Norman, OK 73019, USA

**Keywords:** synchrosqueezing generalized S-transform, time-frequency entropy, hydrocarbon reservoirs detection, random noises

## Abstract

According to the fact that high frequency will be abnormally attenuated when seismic signals travel across reservoirs, a new method, which is named high-precision time-frequency entropy based on synchrosqueezing generalized S-transform, is proposed for hydrocarbon reservoir detection in this paper. First, the proposed method obtains the time-frequency spectra by synchrosqueezing generalized S-transform (SSGST), which are concentrated around the real instantaneous frequency of the signals. Then, considering the characteristics and effects of noises, we give a frequency constraint condition to calculate the entropy based on time-frequency spectra. The synthetic example verifies that the entropy will be abnormally high when seismic signals have an abnormal attenuation. Besides, comparing with the GST time-frequency entropy and the original SSGST time-frequency entropy in field data, the results of the proposed method show higher precision. Moreover, the proposed method can not only accurately detect and locate hydrocarbon reservoirs, but also effectively suppress the impact of random noises.

## 1. Introduction

In the traditional hydrocarbon-reservoir detection, an abnormal attenuation of the high-frequency energy is regarded as an indication of existence of reservoirs. This is because wave induced by fluid flow can lead to stratum absorption, which is the main causation of attenuation of seismic waves [1,2,3,4]. In this case, the energy distribution shows the loss of high-frequency energy and the conservation of strong low-frequency energy [4,5,6,7]. Thus, it is possible to detect reservoirs by comparing the frequency energy distribution [8].

The energy entropy can be used to calculate the change of the energy distribution [9]. When the energy distribution is changed, the entropy will change too. However, the traditional energy entropy is calculated only in the time or frequency domain and cannot detect both time and location of the changes [8,10,11]. Time-frequency analysis methods can well reflect the information of both time and frequency. Thus, some researches have introduced the energy entropy into time-frequency distribution [12,13,14]. Time-frequency entropy uses both time and frequency information to measure any distribution of information and has been widely applied in many practical applications, such as feature extraction and machinery fault diagnosing. In 2009, time-frequency entropy based on S-transform (ST) is applied to reservoir detection by Cai H.P. et al. [15]. The result shows that the greater entropy, the more unstable the frequency. In order to obtain better time-frequency entropy, a high precision time-frequency analysis method is required. Thus, the time-frequency analysis plays a significant role in time-frequency entropy.

Although the traditional time-frequency analysis methods, for instance, short time Fourier transform (STFT) [16], wavelet transform (WT) [17], and S-transform (ST) [18] are widely applied in many areas, and all of them perform well [19,20,21], they still have some disadvantages such as low time-frequency resolution, spectral smearing, the fixed changing trend of the basic wavelet, and so on [20,22,23,24]. These disadvantages limit their application.

Therefore, for better solving those disadvantages, varieties of forms of generalized S transform (GST) on the basis of ST have been proposed by researchers [25,26,27,28,29,30]. In contrast to the ST approach, the generalized S-transform (GST) which is introduced by Gao et al. [27] overcomes the dilemma of the fixed wavelet in ST by introducing four undetermined parameters (amplitude, energy decay rate, energy delay time, and video rate) to construct the basic wavelet adaptive to the non-stationary signal characteristics in practical application.

Inspired by the squeezes along the frequency direction [31,32,33], Chen H. et al. proposes a new high resolution time-frequency analysis method which is named synchrosqueezing generalized S-transform (SSGST) in 2017 [34]. This method introduces GST to replace the WT of synchrosqueezing wavelet (SST) [35,36], squeezes, and reconstructs the complex coefficient spectra of GST results along the frequency direction, so that the energy distributions on the time-frequency spectra are concentrated around the real instantaneous frequency of the target signal and shows a high time-frequency resolution.

In this paper, we propose a novel high-precision time-frequency entropy based on SSGST for reservoir detection. Considering the noise effect, we give a frequency constraint condition to reduce the impact of random noises. The rest of this paper is organized as follows: Section 2 gives the theory of SSGST and proposes the concept of the time-frequency entropy based on SSGST. In Section 3, a synthetic signal is used to demonstrate the high time-frequency resolution of SSGST compared to GST and the time-frequency entropy of two synthetic signals calculated by the proposed method reflects the change of frequency energy distribution of signals. Section 4 applies the field data to verify the high precision of the proposed method in hydrocarbon reservoir detection. Moreover, the proposed method can be useful in suppressing the effect of noise. Lastly, the conclusion of this paper is given in Section 5.

## 2. Materials and Methods

### 2.1. SSGST

In 2017, synchrosqueezing generalized S-transform (SSGST) is proposed by Chen H. et al. [34]. This method based on the generalized S-transform (GST) with four parameters which is proposed by Gao J.H. et al. [27] is defined as:

(1) $$GST_{x}\left(f,b\right)=A\left|f\right|\int_{-\infty }^{\infty }x\left(t\right)\exp\left\{-\alpha {\left[f\left(t-b\right)-\beta \right]}^{2}\right\}\exp\left(-i2\pi f_{0}ft\right)dt$$

where, x(t) is a signal, the basic wavelet amplitude is A, α(α>0) represents the energy attenuation rate. β and $f_{0}$ are energy delay time and video frequency of the basic wavelet, respectively. f is frequency, t is time, and b denotes the time shift.

Then, the instantaneous frequency of the signal can be calculated by:

(2) $$f_{x}(f,b)=f_{0}f+{\left[i2\pi GST_{x}\left(f,b\right)\right]}^{-1}\frac{\partial GST_{x}\left(f,b\right)}{\partial b}$$

Therefore, according to the theories of synchrosqueezing [37] and Function (2), the SSGST is defined as Equation (3):

(3) $$SSGST_{x}\left(f_{l},b\right)=L_{f}^{-1}\sum_{f_{k}:\left|f_{x}\left(f_{k},b\right)-f_{l}\right|\leq \Delta f/2}GST_{x}\left(f_{k},b\right)e^{i2\pi f_{0}f_{k}b}f_{{}_{k}}^{-1}\Delta f_{k}$$

where, $f_{l}$ is the frequency of the result obtained by SSGST. $L_{f}$ denotes the half length of frequency range $\left[f_{l}-L_{f},f_{l}+L_{f}\right]$ centered on the frequency point $f_{l}$. $f_{k}$ represents the discrete frequency points in frequency ranges of the GST, and $\Delta f_{k}=f_{k}-f_{k-1}$.

The Equation (3) represents that the time-frequency spectra values among the frequency range $\left[f_{l}-L_{f},f_{l}+L_{f}\right]$ are superimposed on the frequency point $f_{l}$, so that the SSGST has higher accuracy of time-frequency decomposition ability.

### 2.2. Time-Frequency Entropy Based on SSGST

In this part, we give the basic process of the proposed method named time-frequency entropy based on SSGST. Firstly, performing the SSGST transform to each signal and we can get the time-frequency spectra of signals. Then, calculating the entropy along the time direction based on the time-frequency spectra. For reducing the effect caused by noises in field data, we give an empirical constraint of the frequency, which is presented in Function (4):

(4) $$\left\{ \begin{array}{l} e(f_{i},t_{j})=e(f_{i},t_{j}),f_{low}<f<f_{high}\\ e(f_{i},t_{j})=0,else \end{array}\right.\;i=1,2,\cdots ,N;j=1,2,\cdots ,M$$

where, $e(f_{i},t_{j})$ represents the energy of the *i*-th frequency point at the *j*-th time, and *N* is the number of frequency points, *M* is the number of time points. $f_{low}$ and $f_{high}$ are respectively the lower and upper limit of the effective frequency band of signals. As we all know the frequency distribution of seismic signals is like a normal distribution. The low frequency is from 3 to 10 Hz and the high frequency is usually from 80 to 120 Hz. However, they were determined according to field seismic data quality. In this paper, we give $f_{low}=10$ and $f_{high}=80$.

And the total energy of each time $t_{j}$ is $E_{t_{j}}$:

(5) $$E_{t_{j}}=\sum_{i=1}^{N}e(f_{i},t_{j})$$

Then, the percent of the energy of the *i*-th frequency point is:

(6) p(fi,tj)=e(fi,tj)Etj, i=1,2,⋯,N.

In which $p(f_{i},t_{j})$ is the percent of the energy of the *i*-th frequency point $f_{i}$ at the *j*-th time in the whole signal energy $E_{t_{j}}$ and $\sum_{i=1}^{N}p(f_{i},t_{j})=1$.

So, the time–frequency entropy obtained by the follow formula:

(7) $$S_{t_{j}}=-\sum_{i=1}^{N}p(f_{i},t_{j})\ln p(f_{i},t_{j})$$

**Figure 1:**
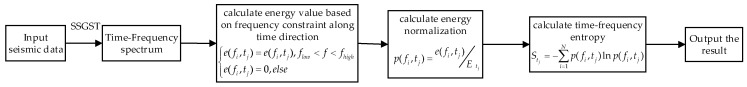
The workflow of the proposed method.

The workflow of the proposed method is shown in Figure 1 and the time-frequency entropy program in this paper is detailed in the supplementary.

## 3. Synthetic Example

In this section, we test the performance of SSGST with a synthetic signal and illustrate the effectiveness of hydrocarbon detection by the proposed method with two different attenuated synthetic signals.

### 3.1. The Time-Frequency Spectra of a Synthetic Signal Using GST and SSGST

**Figure 2:**
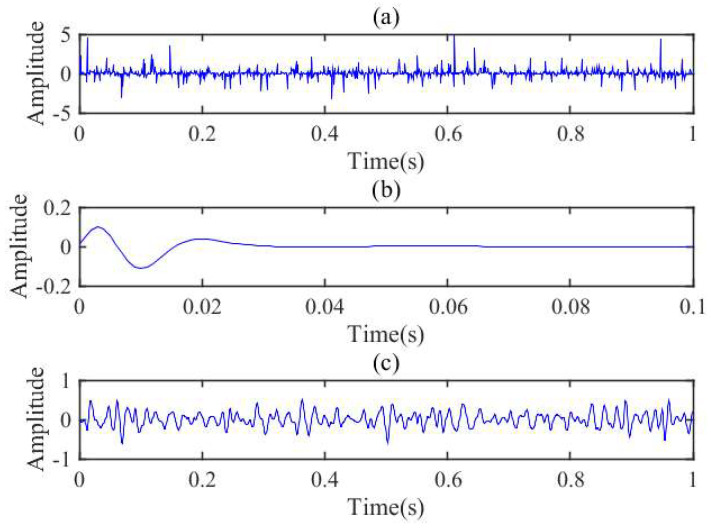
The details of synthetic signal. (**a**) Random reflection coefficients of synthetic signal; (**b**) A minimum phase wavelet with dominate frequency around 60 Hz; (**c**) The synthetic signal generated by convolution of (**a**) and (**b**).

**Figure 3:**
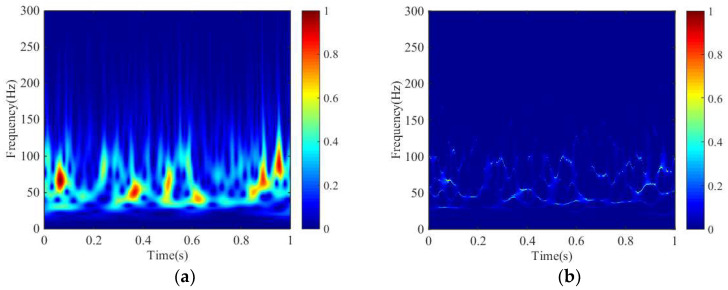
The time-frequency spectra obtained by different time-frequency methods. (**a**) The result obtained by GST; (**b**) The result obtained by SSGST.

In order to show the superior resolution of SSGST better, we design a synthetic signal, which contains 1000 points and the sampling interval is 1 ms, to compare the performance of four-parameter GST and SSGST. Figure 2 shows the details of the synthetic signal. Figure 2a shows random reflection coefficients and a minimum phase wavelet is shown in Figure 2b whose dominant frequency is 60 Hz. Figure 2c is the synthetic signal by convolution of Figure 2a,b. The time-frequency spectra of the synthetic signal (Figure 2c), obtained by SSGST and four-parameter GST, are presented in Figure 3, respectively. From Figure 3a, it can be clearly observed that it is difficult to accurately identify the frequency from 50 to 100 Hz. However, the SSGST method can not only clearly identifies all individual components of the synthetic signal, but also precisely depict frequency of the signal. Therefore, the SSGST can obtain a higher frequency resolution compared to four-parameter GST and due to the high resolution, it can ensure the entropy more accuracy.

### 3.2. The Time-Frequency Entropy of Synthetic Signals

**Figure 4:**
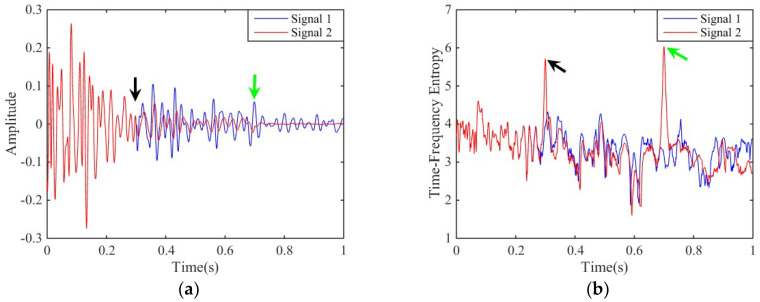
The two synthetic signals and their time-frequency entropy. (**a**) The two synthetic signals with different *Q* values; (**b**) The time-frequency entropy of two synthetic signals.

As we all known, the seismic signal is attenuated during the propagation process and the *Q* value can well simulate the attenuation. Therefore, we use the *Q* value to simulate the seismic attenuation [38]. The *Q* values for the synthetic signal (Figure 1c) are exhibited in Table 1. From Table 1, the synthetic signal is attenuated by different *Q* values. Thus, we get two different attenuated synthetic signals (signal 1 and signal 2). Signal 1 (the blue one in Figure 4a) is attenuated only by one *Q* value (*Q* = 50), and signal 2 (the red one in Figure 4a) is attenuated by three different *Q* values (*Q* = 50, 30, and 20, respectively) in different times.

The two synthetic signals and their time-frequency entropy, calculated by the proposed method, are shown in Figure 4. In Figure 4b, the time-frequency entropy of signal 1 is stable but the time-frequency entropy of signal 2 has two peaks when the *Q* value is changed. The black and green arrows in Figure 4 shows that when the *Q* value turn 50 to 30 and 30 to 20, the time-frequency entropy of signal 2 has increased clearly. Therefore, the entropy value will sharp variation in the attenuation. It is also confirmed that the time-frequency entropy based on SSGST can be used for detecting an abnormal attenuation phenomena of the seismic signals to predict the possibility of underground hydrocarbon reservoirs.

## 4. Field Data

### 4.1. The Time-Frequency Entropy Comparison

**Figure 5:**
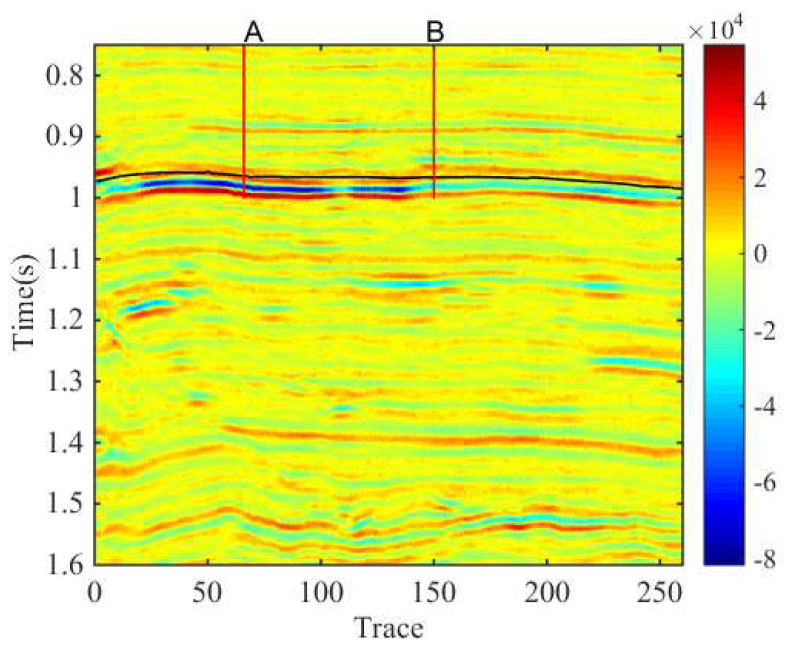
The field seismic data.

In this section, we apply the proposed method to field data, which is from Sichuan Basin, China, to validate the precision of the proposed method in detecting the reservoir. The seismic data consists of 261 traces with 426 sampling points and a sampling interval of 2 ms, the lateral interval is 20 m (Figure 5). In this field data, the well A is a productive well and well B is a non-productive well.

**Figure 6:**
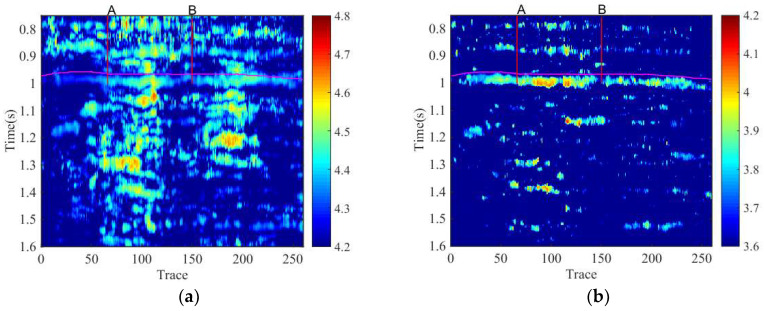
The results obtained by GST time-frequency entropy and the proposed method, respectively. (**a**) The result obtained by time-frequency entropy based on GST; (**b**) The result obtained by the proposed method.

Figure 6a is obtained by GST time-frequency entropy method, and Figure 6b is the result of the proposed method. As can be seen, there is a difference between the entropy values of the reservoir and non-reservoir. Both GST and SSGST have located the hydrocarbon reservoir. However, the proposed method well locates the distribution of the reservoir in time which is consistent with that of field data, and the layer of Figure 6b is better than Figure 6a due to the high precision. Moreover, the time-frequency entropy based on SSGST method has significantly distinguished that the entropy value of well A is bigger than well B, but the results of time-frequency entropy based on GST are not accurate enough.

Therefore, the proposed method seems to be effective in reservoir detection and location. These characteristics make this technique attractive for seismic data processing and interpretation. 

### 4.2. Hydrocarbon Reservoir Detection Performance Analysis with Different Signal-Noise Ratio (SNR)

**Figure 7:**
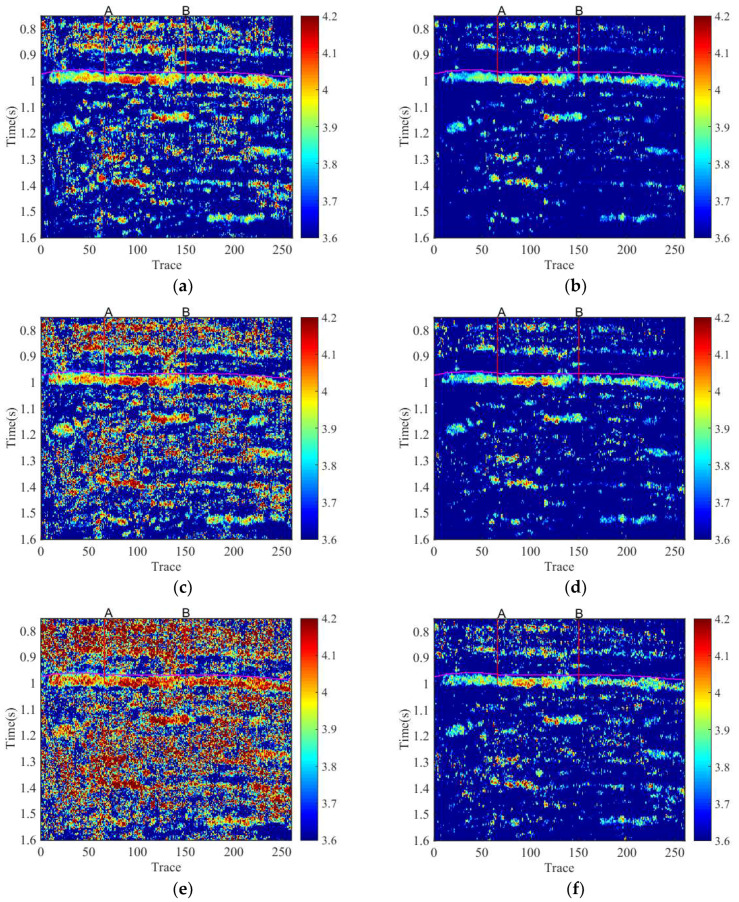
The results obtained by different methods under different SNRs. (**a**) The result of original time-frequency entropy based on SSGST under SNR 35; (**b**) The result of the proposed method under SNR 35; (**c**) The result of original time-frequency entropy based on SSGST under SNR 30; (**d**) The result of the proposed method under SNR 30; (**e**) The result of original time-frequency entropy based on SSGST under SNR 25; and (**f**) The result of the proposed method under SNR 25.

To better understand the sensitivity of the proposed method to the noise level, we have added three different levels of random Gaussian noises into the field data. The SNRs are 25 dB, 30 dB, and 35 dB, respectively. Figure 7 shows the results obtained by original SSGST time-frequency entropy method and the proposed method under different SNRs. All the results show that both of the two methods can detect and locate the hydrocarbon in Figure 7.

Especially, when the SNR reaches 25, the time-frequency entropy spectrum obtained by original SSGST time-frequency entropy in Figure 7e shows that the noise has a heavy impact on entropy calculation, and we cannot clearly distinguish between reservoir and non-reservoir area. However, the proposed method effectively suppresses the random noises, accurately and heuristically identifies the location between reservoir and non-reservoir. 

Thus, the proposed method indeed effectively suppresses the effect of noises and ensure the results more accuracy.

## 5. Conclusions

In this paper, we propose a novel high-precision time-frequency entropy based on SSGST for reservoir detection. By calculating and analyzing the synthetic signals, it is verified that the proposed method can recognize the change of attenuation. Besides, the results of field data confirm that not only the location of the oil and gas layer obtained by the proposed method is the same as the real, but also effectively suppresses the impact of random noises. Therefore, we can conclude that the time-frequency entropy based on SSGST method can be a useful tool for reservoir detection. In practical application, it is necessary to be combined with geological and log data for comprehensive analysis.

## Figures and Tables

**Table 1:** The *Q* value of synthetic signals.

	Time (s)	0–0.3	0.3–0.7	0.7–1
The *Q* Value				
The *Q* value of Signal 1		50	50	50
The *Q* value of Signal 2		50	30	20

## Supplementary Materials

The time-frequency entropy program in this paper is detailed in the supplementary materials online at http://www.mdpi.com/1099-4300/20/6/428/s1.

## References

[1] Xue Y.J., Cao J.X., Tian R.F. (2014). EMD and Teager-Kaiser energy applied to hydrocarbon detection in a carbonate reservoir. Geophys. J. Int..

[2] Matos M.C.D., Marfurt K.J. (2009). Wavelet transform Teager-Kaiser energy applied to a carbonate field in Brazil. Lead. Edge.

[3] Castagna J.P., Sun S., Siegfried R.W. (2003). Instantaneous spectral analysis: Detection of low-frequency shadows associated with hydrocarbons. Lead. Edge.

[4] Xiong X.J., He X.L., Yong P., He Z.H., Kai L. (2011). High-precision frequency attenuation analysis and its application. Appl. Geophys..

[5] Cadoret T., Mavko G., Zinszner B. (1998). Fluid distribution effect on sonic attenuation in partially saturated limestones. Geophysics.

[6] Klimentos T. (1995). Attenuation of P- and S-waves as a method of distinguishing gas and condensate from oil and water. Geophysics.

[7] Winkler U.K., Stuckmann M. (1979). Glycogen, hyaluronate, and some other polysaccharides greatly enhance the formation of exolipase by Serratia marcescens. J. Bacteriol..

[8] Tavakkoli F., Teshnehlab M. A ball bearing fault diagnosis method based on wavelet and EMD energy entropy mean. Proceedings of the International Conference on Intelligent and Advanced Systems.

[9] Yang Y., Yu D., Cheng J. (2006). A roller bearing fault diagnosis method based on EMD energy entropy and ANN. J. Sound Vib..

[10] Peng Z., Chu F., He Y. (2002). Vibration signal analysis and feature extraction based on reassigned wavelet scalogram. J. Sound Vib..

[11] Li C.J., Wu S.M. (1989). On-Line Detection of Localized Defects in Bearings by Pattern Recognition Analysis. J. Eng. Ind..

[12] Yu D.J., Yang Y., Cheng J.S. (2007). Application of time-frequency entropy method based on Hilbert-Huang transform to gear fault diagnosis. Measurement.

[13] Ren W.-X., Sun Z.-S. (2008). Structural damage identification by using wavelet entropy. Eng. Struct..

[14] Huang N., Chen H., Zhang S., Cai G., Li W., Xu D., Fang L. (2015). Mechanical Fault Diagnosis of High Voltage Circuit Breakers Based on Wavelet Time-Frequency Entropy and One-Class Support Vector Machine. Entropy.

[15] Cai H.P., He Z.H., Huang D.J., Li R. (2010). Reservoir Distribution Detection based on Time-Frequency Entropy. J. Oil Gas Technol..

[16] Allen J. (2003). Short term spectral analysis, synthesis, and modification by discrete Fourier transform. IEEE Trans. Acoust. Speech Signal Process..

[17] Daubechies I. (1992). Ten Lectures on Wavelets.

[18] Stockwell R.G., Mansinha L., Lowe R.P. (1996). Localization of the Complex Spectrum: The S Transform. IEEE Trans. Singal Process..

[19] Avargel Y., Cohen I. (2007). System Identification in the Short-Time Fourier Transform Domain with Crossband Filtering. IEEE Trans. Audio Speech Lang. Process..

[20] Mallat S.G. (2009). A Wavelet Tour of Signal Processing, Third Edition: The Sparse Way.

[21] Dash P.E.K., Panigrahi B.K., Panda G. (2007). Power Quality Analysis Using S-Transform. IEEE Power Eng. Rev..

[22] Sun S. (2002). Examples of wavelet transform time-frequency analysis in direct hydrocarbon detection. Seg Tech. Program Expand. Abstr..

[23] Gilles J. (2013). Empirical Wavelet Transform. IEEE Trans. Signal Process..

[24] Mandic D.P., Rehman N.U., Wu Z., Huang N.E. (2013). Empirical Mode Decomposition-Based Time-Frequency Analysis of Multivariate Signals: The Power of Adaptive Data Analysis. Signal Process. Mag. IEEE.

[25] Mcfadden P.D., Cook J.G., Forster L.M. (1999). Decomposition of gear vibration signals by the generalized S transform. Mech. Syst. Signal Process..

[26] Pinnegar C.R., Mansinha L. (2003). The S-transform with windows of arbitrary and varying shape. Geophysics.

[27] Gao J.H., Chen W.C., Youming L.I., Tian F. (2003). Generalized S Transform and Seismic Response Analysis of Thin Interbedss Surrounding Regions by Gps. Chin. J. Geophys..

[28] Sejdić E., Djurović I., Jiang J. (2007). A Window Width Optimized S-Transform. EURASIP J. Adv. Signal Process..

[29] Chen X.H., He Z.H., Huang D.J., Wen X.T. (2009). Low frequency shadow detection of gas reservoirs in time-frequency domain. Chin. J. Geophys..

[30] Li D., Castagna J., Goloshubin G. (2016). Investigation of generalized S-transform analysis windows for time-frequency analysis of seismic reflection data. Geophysics.

[31] Mousavi S.M., Langston C.A., Horton S.P. (2016). Automatic microseismic denoising and onset detection using the synchrosqueezed continuous wavelet transform. Geophysics.

[32] Mousavi S.M., Langston C.A. (2017). Automatic noise-removal/signal-removal based on general cross-validation thresholding in synchrosqueezed domain and its application on earthquake data. Geophysics.

[33] Auger F., Flandrin P., Lin Y.T., Mclaughlin S., Meignen S., Oberlin T., Wu H.T. (2013). Time-Frequency Reassignment and Synchrosqueezing: An Overview. IEEE Signal Process. Mag..

[34] Chen H., Lu L.Q., Xu D., Kang J.X., Chen X.P. (2017). The Synchrosqueezing Algorithm Based on Generalized S-transform for High-Precision Time-Frequency Analysis. Appl. Sci..

[35] Duchesne M.J., Halliday E.J., Barrie J.V. (2011). Analyzing seismic imagery in the time-amplitude and time-frequency domains to determine fluid nature and migration pathways: A case study from the Queen Charlotte Basin, offshore British Columbia. J. Appl. Geophys..

[36] Daubechies I., Lu J., Wu H.T. (2011). Synchrosqueezed wavelet transforms: An empirical mode decomposition-like tool. Appl. Comput. Harmon. Anal..

[37] Wu H., Flandrin P., Daubechies I. (2011). One or two frequencies? The synchrosqueezing answers. Adv. Adapt. Data Anal..

[38] Li F., Zhou H., Jiang N., Bi J., Marfurt K.J. (2015). Q estimation from reflection seismic data for hydrocarbon detection using a modified frequency shift method. J. Geophys. Eng..

