# Supplementary material for: A High-Precision Time-Frequency Entropy Based on Synchrosqueezing Generalized S-Transform Applied in Reservoir Detection
Source: Entropy (Basel). 2018 Jun 3;20(6):428. doi: 10.3390/e20060428 (PMC7512946; doi:10.3390/e20060428)
Supplement: Supplementary file 1 [file entropy-20-00428-s001.pdf]

The time-frequency entropy code in this paper is written based on MATLAB.

```
function Entropy = sstfe(TFdata,fs,f1,f2)
%%
% INPUT:
% TFdata：Time-frequency speatrum data
% fs：sampling frequency
% f1：The initial frequency of the constraint frequency
% f2：The cut-off frequency of the constraint frequency
% OUTPUT:
% Entropy: The output time frequency entropy

%%
Entropy = [];

[M,N] = size(TFdata);
start_fre = f1;
end_fre = f2;
M1 = floor(start_fre/M * fs);
M2 = floor(end_fre/M * fs);
%%
if M > N
    TFdata = TFdata';
end
%%
for i = 1:N

    timetrace = TFdata(:,i);
%%
    for j = 1:M1-1

        timetrace(j) = 0;
    end
    for j = M2+1:M;

        timetrace(j) = 0;
    end
%%
    E = sum(timetrace);

    if E == 0
        s = 0;
    else
        for k = 1 : M
```

```
            q(k) = timetrace(k)/E;
            if q(k) == 0 && E~=0;
                En(k) = 0;
            else
                En(k) = - q(k) * log(q(k));
            end
        end
        s = sum(En);
    end
    Entropy = [Entropy,s];
end
```
